# Identification of ACE I-Inhibitory Peptides Released by the Hydrolysis of Tub Gurnard (*Chelidonichthys lucerna*) Skin Proteins and the Impact of Their In Silico Gastrointestinal Digestion

**DOI:** 10.3390/md21020131

**Published:** 2023-02-17

**Authors:** Hajer Bougatef, Cristina de la Vega-Fernández, Assaad Sila, Ali Bougatef, Oscar Martínez-Alvarez

**Affiliations:** 1 Laboratory for the Improvement of Plants and Valorization of Agroresources, National School of Engineering of Sfax (ENIS), University of Sfax, Sfax 3038, Tunisia; 2 Institute of Food Science, Technology and Nutrition (ICTAN, CSIC), 6 José Antonio Novais St, 28040 Madrid, Spain; 3 Department of Life Sciences, Faculty of Sciences of Gafsa, University of Gafsa, Gafsa 2100, Tunisia

**Keywords:** by-catch, protein hydrolysates, angiotensin converting enzyme, bioactive peptides, upgrading, fish skin, tub gurnard, functional ingredient, *Chelidonichthys lucerna*

## Abstract

Tub gurnard is a highly abundant fishery species caught as a discard in the Mediterranean Sea. This work proposes its valorisation through the release of potential antihypertensive peptides and glycosaminoglycans (GAGs) through the controlled hydrolysis of tub gurnard skin proteins. Four proteases (Esperase, Alcalase, Trypsin and Pronase E) were used to obtain potent angiotensin converting enzyme I (ACE)-inhibitory hydrolysates. Peptides and GAGs were separated and evaluated for their antihypertensive potential by fluorometry. The peptide-rich fractions derived from the Esperase and Alcalase hydrolysates showed very low IC_50_ values (47 and 68 μg/mL, respectively). Only the GAGs from the Trypsin and Esperase hydrolysates were relevant ACE inhibitors (63 and 52% at 1 mg/mL, respectively). The peptide composition of the most potent ACE-inhibitory fractions derived from the Esperase and Alcalase hydrolysates (IC_50_ values of 33 and 29 μg/mL, respectively) was analysed by RP-LC-ESI-MS/MS. The analysis suggests that the ACE-inhibitory activity is related to the peptide hydrophobicity, as well as to the presence of specific residues at any of the last four C-terminal positions. The in silico gastrointestinal digestion of these fractions yielded small peptides with antihypertensive potential.

## 1. Introduction

In the past 30 years, the number of people aged 30–79 with hypertension increased from 650 million to 1.28 billion. Almost half of these people (580 million) did not know they had the disease [1]. For the global medical community, preventing and treating hypertension has grown to be a challenging feat. Although hypertension is easily diagnosed and can be controlled with inexpensive drugs, many people still do not receive the attention they need, particularly in sub-Saharan Africa, South, Central and South-East Asia, and the Pacific Island countries. The use of synthetic, angiotensin-converting enzyme (ACE) inhibitors is considered the first-choice therapy [2]. However, due to their adverse effects, finding natural ACE inhibitors is of high interest for the prevention and management of moderate hypertension [3,4], especially if they can be incorporated into foods as ingredients. In this regard, the main interest has focused on identifying natural sources of ACE inhibitors such as spent hen [5], microalgae [4], fish and fish processing by-products [3,6]. Peptides released by the controlled hydrolysis of proteins are the most-studied natural ACE inhibitors. The protein, which is used as a raw material, can come from a wide variety of natural sources, i.e., fish by-products and underutilized marine species, as reviewed by Phadke et al. [7].

Trawl fishing has been studied in the past few decades due to the high number of species that are caught accidentally; these are called bycatch. The main reasons for discarding fish are low or null commercial interest, regulations and low quality of the catch. Minimizing bycatch in fisheries has been seen as an emerging issue, stimulating the search for possible solutions [8]. In this regard, the European Parliament published regulation (EU) No 1380/2013, which obliges the landing of discarded species, thus leading fisheries to be much more selective with their catches. However, in reality, large quantities of discarded species are landed daily in European ports. Shrimp trawl fisheries produce the highest amount of discards, while trawl fisheries, and especially artisanal fisheries, have a considerably lower discard rate. However, the huge presence of artisanal fleets in some areas, such as the Mediterranean, makes the total amount of discards caught very important [9]. Discards are mainly destined for the production of low-value fishmeal, disposed of as waste or used as fertilizer, making it necessary to devise new ways of valorising this abundant raw material, which is rich in high-quality nutrients.

The use of proteases to hydrolyse this raw material under controlled conditions to obtain bioactive peptides would maximize the economic return while contributing to environmental sustainability [10]. These peptides could be included as ingredients in high-value-added functional foods, conferring upon them diverse functions such as antihypertensive, hypoglycaemic, nootropic, antioxidant and antiproliferative functions [11]. The processing parameters will have a significant impact on the bioactive properties of the released peptides (enzyme specifications, hydrolysis temperature and duration, raw material, etc.), as reported by Sarmadi et al. [12]. Moreover, discards could be a source of vitamins, minerals, polyunsaturated fatty acids and polysaccharides (mainly GAGs such as chondroitin sulphate, hyaluronic acid or dermatan sulphate), all with bioactive properties of interest for human health [11,13]. Considering their capacity to interact with several proteins, sulphated glycosaminoglycans have several biological activities, making them important drugs for use in clinical and pharmaceutical fields.

The tub gurnard (*Chelidonichthys lucerna*) is an abundant coastal species distributed along the Mediterranean and Black Seas, and the eastern Atlantic coast. Tub gurnard is caught as a discard in the Mediterranean. It is usually thrown back into the sea or directly brought to port and marketed, at a very low price, for the preparation of soups. Although tub gurnard is rarely commercialized in fillets, its flesh has high nutritional value [14] and could be used for the production of restructured products in a process that would result in the generation of various by-products, such as skins.

The development of an environmentally friendly bioconversion process that leads to the release of bioactive compounds from tub gurnard would be of great interest for its valorization. In this line, the main objective of this work is to obtain and identify potential antihypertensive peptides from tub gurnard skins through controlled protein hydrolysis, using proteases and not utilizing a previous protein extraction step. A second objective of this work is to evaluate the potential antihypertensive capacity of the skin GAGs with the intention to promote in-depth research and the sustainable use of tub gurnard skins.

## 2. Results and Discussion

### 2.1. Chemical Analysis of Raw Material

The tub gurnard (*Chelidonichthys lucerna*) skins had a significant protein content (31 ± 3%) within the range (8 to 35%) of protein present in different fish by-products [15]. The moisture and ash contents were 59 ± 1% and 9 ± 1%, respectively. The amino acid composition of the raw material, expressed as residues/1000 residues, was also determined. Gly was the most abundant amino acid (Table 1), accounting for approximately 30% of the total amino acids, followed by Ala (111/1000), Pro (80/1000) and Hyp (54/1000). These residues are abundant in fish collagens [16]. According to the literature, the general structure of the collagen monomer is Gly-X-Y, in which X and Y are often Pro and Hyp, respectively. The imino acid (Pro and Hyp) content in the raw material was similar to the imino acid content described in other fish skins; however, it was lower than the amount present in mammalian collagen [17]. In general, the different Pro and Hyp contents in collagen are mainly related to different living environments and habitat temperatures. The raw material was also abundant in acidic or amide-containing residues (Asp + Asn and Glu + Gln), while very low Cys, Tyr, His and Hyl contents were found. The essential amino acid content was moderate, while the hydrophobic amino acid content was particularly relevant; this is of interest, given that fish by-products with high amounts of aromatic and hydrophobic amino acids are a good source of peptides with antihypertensive potential [4].

### 2.2. Degree of Hydrolysis (DH)

The DH increased rapidly during the first hour (data not shown). Thereafter, the DH gradually decreased (when Alcalase or Esperase were used) or remained constant. The decrease in the rate of hydrolysis was the result of a reduction in the number of susceptible peptide bonds and/or enzyme inactivation for different reasons, such as the release of inhibitory peptides [7]. Esperase showed the highest hydrolytic capacity, reaching a final DH value of 11.99%. Alcalase and Trypsin also showed a high capacity for hydrolysing skin protein but scored lower than Esperase, attaining DH values of 9.13% and 7.74%, respectively. The highest DHs obtained using Esperase and Alcalase would be the result of their broad specificity. Both are serine endo-proteases that show not only peptidase activity but esterase and amidase activities as well. Trypsin is an endopeptidase that only cuts in the carboxyl side of Lysine or Arginine, which could explain the lower DH attained with this enzyme. Although Pronase E is a mixture of proteolytic enzymes with the ability to hydrolyse most peptide bonds, it demonstrated the lowest skin protein hydrolytic capacity, reaching a DH value of only 5.60%. This could be due to the optimal activity of this enzyme at a higher (8.8) pH than the pH of known alkaline proteases (8.6) or even a high instability under the conditions used for hydrolysis.

### 2.3. MW Distribution of the Hydrolysates

The MW distribution is an important parameter influencing the biological and technological properties of protein hydrolysates. The Pronase E hydrolysate was mainly composed of polypeptides (3.7 kDa), which is consistent with the low DH it achieved (Table 2). This would limit the use of this hydrolysate as a functional ingredient, given the difficulty the high-MW peptides have in crossing the intestinal barrier. The Trypsin hydrolysate, with an average molecular weight of 2.05 kDa, would have the same limitation as a functional ingredient. In contrast, the Esperase and the Alcalase hydrolysates would be much more suitable as functional ingredients due to the abundance of low-MW peptides (<1 kDa), which is the result of the extensive hydrolysis carried out by these enzymes due to their highly non-specific protein cleavage. The MW profile observed at 280 nm showed a high content of aromatic peptides of very low molecular weight (0.35–0.54 kDa) in the Esperase and Alcalase hydrolysates, presumably di- and tripeptides, whereas the Trypsin hydrolysate was rich in aromatic peptides of approximately 1.6 kDa. The Pronase E hydrolysate also contained high-MW aromatic peptides (2.16 kDa).

### 2.4. ACE-Inhibitory Activity

All the protein hydrolysates exhibited a potent ACE-inhibitory ability, demonstrating inhibiting values higher than 86% at a final concentration of 1 mg/mL (Table 2). The Esperase hydrolysate exhibited the highest ACE-inhibiting activity (93.92% at 1 mg/mL), followed by the Trypsin hydrolysate (90.92%) at the same concentration. The ACE-inhibitory ability of the Alcalase hydrolysate was slightly but significantly lower than that of the Trypsin hydrolysate, and statistically similar to the ACE-inhibitory ability of the Pronase E hydrolysate. Byun and Kim [18] also found similar ACE-inhibitory activity of Alcalase- and Pronase E-derived Alaska Pollock skin protein hydrolysates. The slight differences in the inhibitory capacity of the samples would be attributed to the specific characteristics of the released peptides, including the amino acid sequence, length, charge and hydrophobicity [19]. These characteristics will depend on both the enzyme used and the DH, which will be higher or lower following the temperature, pH and hydrolysis time used. Thus, different studies have shown a positive correlation between DH and ACE-inhibitory activity. This is consistent with the findings of Nuchprapha et al. [20], which observed the highest ACE-inhibiting activity in the smallest fraction (MW < 1 kDa) of a longan seeds protein hydrolysate.

The sulphated GAG content of the Esperase, Alcalase and Trypsin hydrolysates was determined, reaching values of 157 ± 3, 136 ± 8 and 104 ± 9 mg of chondroitin 4-sulfate equivalents/g of dried hydrolysate, respectively. The GAGs (sulphated and non-sulphated) were separated from the peptides, and the ACE-inhibitory activity of both fractions was analysed. All GAG-rich fractions showed ACE-inhibitory activity, though to a lesser extent than the hydrolysates and the PRFs (Table 3). The GAGs derived from the Trypsin hydrolysate showed the highest inhibiting activity (63%), followed by those from the Esperase hydrolysate. In contrast, the GAGs derived from the Alcalase hydrolysate hardly inhibited ACE. These results, together with the GAG content of the hydrolysates, implied that the ACE-inhibitory activity of the GAG-rich fractions was not only related to their content. In this regard, GAGs are released during protein hydrolysis; however, proteases cannot hydrolyse the glycosidic bond between the linker tetrasaccharide and the serine hydroxyl groups in the protein core [21]. This implies that the protein hydrolysis releases GAGs with short, covalently linked amino acid sequences that would play a relevant role in ACE-inhibitory potency.

Only a few reports have focused on the ACE-inhibitory activity of marine GAGs. The ACE-inhibitory activity of GAGs from tub gurnard skin was slightly lower than what was reported by Karimzadeh [22] for GAGs extracted from sturgeon cartilage (85.7% ACE inhibition at a concentration of 1 mg/mL), and by Krichen et al. [23] for GAGs from Atlantic Bluefin tuna skin (70.81% at a concentration of 0.8 mg/mL). The ACE-inhibitory activity was also lower than the values reported by Abdelmalek et al. [24] for GAGs released from common squid skin (50% at 0.14 mg/mL).

The MW profile of the peptide-rich fractions (PRF) was similar to that of the protein hydrolysates (data not shown), indicating that the GAG isolation process did not affect the peptide composition. All PRFs showed a very potent ACE-inhibitory activity, achieving low IC_50_ values between 47 and 89 μg/mL (Table 3). The Esperase PRF exhibited the most potent ACE inhibiting activity, followed by the Alcalase PRF and the Trypsin PRF. These results agreed with the work of Mosquera et al. [25], who reported that Esperase is more effective at releasing ACE-inhibitory peptides from giant squid protein than Alcalase. Previous studies conducted on codfish blood and sardine [26] showed that Alcalase is more effective at producing potent ACE-inhibitory hydrolysates than Trypsin and Pepsin. The small differences between the IC_50_ values could be attributed to the presence of specific peptides of different sequences and sizes in the hydrolysates; the presence of these peptides is related to the DH. The IC_50_ values obtained were much lower than those of Alcalase, Neutrase or Flavourzyme protein hydrolysates from dark tuna meat (0.24–0.31 mg/mL), as reported by Mongkonkamthorn et al. [27]. In addition, Heffernan et al. [28] reviewed the ACE-inhibitory activity of more than 20 fish protein hydrolysates, none of which showed a higher activity than the tub gurnard skin hydrolysates. Similarly, the IC_50_ values were lower than those found by Wijesekara and Kim [29] for different non-purified seafood protein hydrolysates. Mosquera et al. [25] reported a similar ACE-inhibitory activity (51–93 µg of protein/mL) of Alcalase and Esperase protein hydrolysates from the tunic of a giant squid, although they were previously fractionated to concentrate the peptides responsible for this activity.

### 2.5. Isolation and Purification of ACE-Inhibitory Peptides

The Alcalase and Esperase PRFs were selected for the isolation of ACE-inhibitory peptides. They were fractionated using HPLC, yielding five (A_I_–A_V_) and four fractions (E_I_-E_IV_), respectively (Figure S1). The ACE-inhibiting activity of all these fractions was tested (Table S1). A_I_ showed the lowest activity (36%), and A_IV_ demonstrated the highest activity (84%). Av also exhibited a high ACE-inhibiting activity of approximately 86%.

The fractions derived from Esperase PRFs also exhibited a very high ACE-inhibitory capacity (84–93%, Table S1), with E_IV_ being the most effective fraction. The different ACE-inhibiting activities of the fractions could be ascribed to different peptide compositions, charges, hydrophobicity and size.

The most potent ACE-inhibitory fractions (A_IV_, A_V_ and E_IV_) were fractionated by chromatography (Figure S2), and the ACE-inhibitory activity of all the sub-fractions was determined (Table S2). The most potent ACE-inhibiting sub-fractions (A_IV-5_ and E_IV-1_) showed very similar IC_50_ values (29 and 33 µg/mL, respectively) and lower values than that of the PRFs, indicating that the ACE-inhibiting peptides were successfully concentrated by chromatography. Nonetheless, it is important to note that other fractions also contained ACE-inhibiting peptides. A_IV-5_ and E_IV-1_ were selected, and their peptide composition was analysed by RP-LC-ESI-MS/MS. 

### 2.6. Identification of the Peptide Composition of the Most Potent ACE-Inhibiting Fractions

Seventy-one peptides were identified in A_IV-5_. Sixty-one were against the acceptance criteria (de novo score ≥ 80, −10lgP ≥ 20). Thirty-three of these peptides were deduced from mass spectral fragmentation data (de novo sequenced) and considered novel peptides. Among them, ten demonstrated ALC scores higher than 90%, although the relative abundance of most of these peptides in the fraction was negligible. The peptides in the fraction showed a molecular mass between 693 and 1952 Da (6–22 residues), and most of them included some hydroxyproline residue. Most of the peptides lower than 1100 Da (38 of 49) were novel peptides. Thirteen peptides, of which nine were novel (Table 4), constituted 89% of the total except for the three most abundant peptides (72% of the total) These peptides were identified as GPPGS(sub P)PGPAGPP(+15.99)GPPGSGM (37.5%, 1586 Da), GM(+15.99)P(+15.99)GERGAAGLP(+15.99)GLR (24.6%, 1486 Da) and GPAGIVGPP(+15.99)GPAGPA (9.9%, 1230 Da). According to the Uniprot database, the three peptides matched with the collagen sequences of different fish species. The most abundant peptide corresponded to a fragment of the chain-like collagen alpha-1(I), which was described for *Lamprologus brichardi* (family Gobiidae). The three peptides showed a high MW and were composed of more than ten residues. According to Shi et al. [30], these major peptides were not the main enzyme inhibitors due to the inability of the ACE active site to accommodate high-MW peptides It seems to be that non-abundant and shorter peptides were mainly responsible for ACE inhibition.

Ninety-six peptides were identified in E_IV-1_. Eighty-eight of them were against the acceptance criteria. Sixty-nine of these peptides were novel peptides, with twenty-five of them showing an ALC score higher than 90%. Twenty-nine peptides demonstrated a sequence that matched with collagen sequences from different fish species; this is logical, given that collagen is the main protein in the raw material used. Their molecular mass ranged between 3128 and 678 Da, and they were composed of between 34 and 5 residues. Most of the peptides lower than 1500 Da were novel peptides. The peptides that showed the highest relative abundance were LLAPPERKY (30.8%, 1085 Da), ELEEELEAE (15.4%, 1089 Da) and GPRGPAGPL (11.9%, 820 Da). The latter matched with collagen sequences of numerous fish species (Table 5), while the others were novel peptides.

None of the peptides in Table 4 and Table 5 have been described as ACE inhibitors. Some peptides sharing a similar sequence were found in both fractions, such as the collagen-derived peptides AGP(+15.99)PGFP(+15.99)GGP(+15.99)GPKGEIGPA and AGPP(+15.99)GFP(+15.99)GGPGP(+15.99)KGELGPA. The ACE-inhibitory capacity of collagen-derived peptides (in vitro and in vivo) was described by Cao et al. [31], and some of them could be responsible for the ACE-inhibitory activity of the fractions. However, the high MW of most of the collagen-derived peptides present in the fractions (higher than 1 kDa) and their high number of residues (more than 10) suggest that they were not primarily responsible for the enzyme inhibition. Therefore, the ACE-inhibiting activity would be due to the presence of shorter and less abundant peptides in the active fractions: mainly new peptides.

Peptides can inhibit ACE activity by competitive, uncompetitive and non-competitive mechanisms. However, most seafood-derived ACE-inhibitory peptides are known to be competitive inhibitors, which can bind either to the active site and block it or to the binding site away from the active site, altering the enzyme conformation and affecting substrate binding to the active site. The binding to ACE seems to be strongly influenced by the C-terminal sequence of the peptides. Wang et al. [32] reported that the presence of hydrophobic amino acids (aromatic or branched-chain as H, Y, F, W, L, I or V) at three C-terminal positions is a major determinant of binding ACE. In A_IV-5_, 43 out of 71 peptides (42.8% of the total) demonstrated any of these residues in these positions, whereas in E_IV-1_, they were 65 out of 96 (71%). Similarly, and according to the last authors, the positive charge of K and R increases the ACE-inhibiting potency. These residues were found at the C-terminal positions in ten peptides (44.1%) in A_IV-5_, including the second and fourth most abundant. Eighteen peptides in E_IV-1_ presented K or R in the C-terminal position. In a similar study, Wu et al. [33] reported that specific residues in the last four C-terminal positions of long-chain peptides (4–10 residues) could directly interact with ACE active sites. These authors observed that the presence of Y or C at the C-terminus was associated with the highest ACE-inhibitory activity, followed by H, W or M at the penultimate position, I, L, V and M at the antepenultimate, and W at the next position. In A_IV-5_, Y was found at the C-terminus of eight peptides, and L, V and M were found at the antepenultimate C-terminal position of 7, 2 and 2 peptides, respectively. Similar results were observed in E_IV-1_, with the presence of L, V and M at the penultimate C-terminal position of 9, 4 and 3 peptides, respectively. Therefore, the presence of L at that position, as well as the presence of Y at the C-terminus, could play a relevant role in the ACE-inhibitory capacity. However, it is important to note that most of the peptides with residues at these positions attained ALC values between 80 and 90 %. Furthermore, Wijesekara and Kim [29] reported that the presence of hydrophobic amino acids (A, L, I, V, P, F, W and M) at the amino-terminal end is common in ACE-inhibitory peptides, as is the case with LLAPPERKY.

These results suggest that the high inhibitory capacity of the analysed fractions was probably due to the concomitant action of different peptides, attributed to the presence of specific residues at certain positions of the sequence; for example, K or R at the C-terminus, and hydrophobic residues at mainly three positions of the C-terminus [32,34] and at the N-terminus.

### 2.7. In Silico Gastrointestinal Digestion of the Peptides in the Most Potent ACE-Inhibiting Fractions

According to the BIOPEP-UWM tool, the simulated gastrointestinal digestion of A_IV-5_ released numerous peptides with bioactive capacity. Seven of these, mostly dipeptides, have been described as ACE-inhibitory peptides (Table 6). The most abundant peptides were DF, VM and PGL.

According to the BIOPEP database, the in silico digestion of E_IV-1_ yielded 45 bioactive peptides. Among them, 13 peptides (mostly composed of two residues) were identified as ACE-inhibitory peptides. PL and GY were the most abundant (Table 7).

These results indicate that all peptides present in the bioactive fractions are susceptible to being hydrolysed during gastrointestinal digestion and therefore require protection by micro- or nano-encapsulation. Encapsulation by specific matrices would also help bioactive peptides to cross the intestinal barrier and reach the target organs intact. However, it is important to note that gastrointestinal digestion could be of interest to release small ACE-inhibitory peptides that could pass directly through the intestine.

## 3. Materials and Methods

### 3.1. Reagents

Alcalase^®^ 2.4 L (from *Bacillus licheniformis*), Pancreatic Trypsin Novo 6.0S, Protease type XIV, also known as Pronase E (from *Streptomyces griseus*) and Esperase^®^ 8.0 L (from Bacillus spp.) were obtained from Novozymes (Bagsværd, Denmark). Angiotensin-Converting Enzyme I (ACE, EC 3.4.15.1), aprotinin, vitamin B12, angiotensin II, hippuryl histidyl leucine (HHL) and glycine were obtained from Sigma Chemical Co., (St. Louis, MO, USA). Abz-GLY-PHe(NO_2_)-Pro was obtained from Bachem Feinchemikalien (Bubendorf, Switzerland). All other chemicals and reagents used were of analytical grade and were purchased from Panreac Chemical Corp. (Barcelona, Spain).

### 3.2. Sample Preparation

The tub gurnards (*Chelidonichthys lucerna*) were captured as bycatch by a fishing boat off of the Mediterranean coast. They were purchased at the local market immediately after landing. They were packed in polyethylene bags, covered with ice, and transported to Madrid (Spain). The tub gurnards were beheaded manually, and the skin, bones and muscles were separated using a Baader 964 machine (BAADER Food Processing Machinery, Lübeck, Germany). The skins were treated with acetone (skin:acetone ratio of 1:3, *w*:*v*), for 2 h to remove pigments and lipids, rinsed twice with distilled water, dried at 45 °C, and stored at 4 °C until used.

### 3.3. Proteolytic Activity

The proteolytic activity was measured according to Lajmi et al. [35], using casein as a substrate. One unit of proteinase activity (1 U) was defined as the amount of enzyme required to release 1 μg of tyrosine per minute under the experimental conditions used.

### 3.4. Analysis of the Proximate Composition of the Raw Material

AOAC standard techniques 930.15 and 942.05 [36] were used to determine the moisture and ash content of the raw material, respectively. The protein content was determined from the total nitrogen content by the Dumas method (AOAC 992.15). A LECO TruMac^®^ N analyser (Leco Corp., AG Geleen, The Netherlands). A conversion factor of 6.25 was used to calculate the protein content.

### 3.5. Extraction Procedure

#### 3.5.1. Preparation of Enzymatic Hydrolysates

Before starting hydrolysis, 40 g of dried skins was heated at 95 °C in boiling water for 10 min to inactivate endogenous enzymes. After cooling, they were homogenized in 200 mL of Tris HCl buffer (0.2 M, pH 8.6). The protein hydrolyses were performed under the controlled conditions of the pH and temperature indicated in Table 2. The enzyme was added in an enzyme/protein ratio of 3 U/g of protein. During the hydrolysis process, the temperature was controlled, and the pH was adjusted to the initial value with 1 M NaOH, using a pH-Stat Titromatic 1S (Crison Instruments, Barcelona, Spain). After 2 h, the protein hydrolysates were heated for 15 min at 80 °C to inactivate the enzyme. They were then centrifuged at 15,000× *g* for 20 min (Beckman Coulter J2-mc, Indianapolis, IN, USA). The supernatants were freeze-dried and stored at −20 °C for further use.

#### 3.5.2. Separation and Purification of Glycosaminoglycans

The GAGs were extracted after protein hydrolysis, according to Ben Mansour et al. [37]. Firstly, 1% (*w*/*v*) of cetylpiridinium chloride was added to the hydrolysates. The mixtures were kept for 24 h at room temperature and then centrifuged at 5000× *g* for 20 min, also at room temperature. The supernatants (peptide-rich fractions: PRFs) were collected and freeze-dried, while the precipitates containing GAGs were suspended in 50 mL of 2 M of NaCl with ethanol (100:15, *v*/*v*) at 4 °C overnight to remove the cetylpyridinium chloride (CPC). Thereafter, they were centrifuged at 5000× *g* for 30 min at 4 °C. The precipitates were washed with 80% ethanol, then with absolute ethanol, and were finally evaporated using a rotary evaporator. Finally, the dried fractions were dissolved in distilled water and freeze-dried to obtain the GAGs.

**Figure 1:**
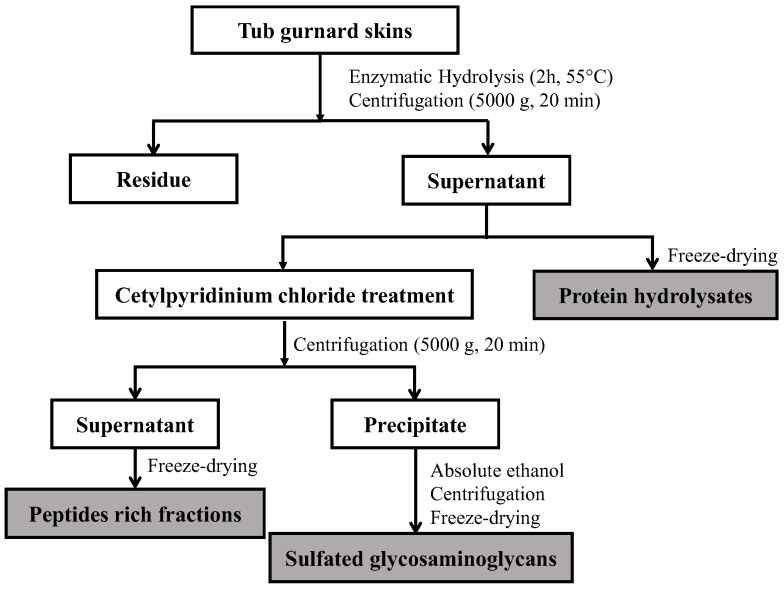
Procedure for fractionation and separation of bioactive fractions from tub gurnard skins.

A diagram of the process followed to obtain the peptide-rich fractions and the GAGs from the tub gurnard skins is shown in Figure 1.

### 3.6. Determination of the Degree of Hydrolysis

The degree of hydrolysis (DH) was calculated according to Adler-Nissen [38]. The total number of peptide bonds (htot) used was 10.36 mEq/g.

### 3.7. Determination of the Molecular Weight (MW) Profile

The MW profiles were obtained according to Martínez-Alvarez et al. [39], using a Superdex peptide PC 3.2/30 column (GE Healthcare, Barcelona, Spain, fractionation range 7000–100 Da). The solvent used was 30% (*v*/*v*) acetonitrile/Milli-Q water (Millipore Co., New Bedford, MA, USA) with 0.1% (*v*/*v*) TFA. The flow rate was 0.1 mL/min. First, the dry powders were dissolved in Milli-Q water at a concentration of 10 mg/mL, centrifuged at 9000× *g* for 5 min, and filtered using 0.45 µm syringe filters. The absorbance was measured at 214 and 280 nanometres. The height of the peaks was used to calculate the relative composition.

### 3.8. Analysis of Amino Acid Compositions

The amino acid composition was determined using ion exchange chromatography with post-column ninhydrin derivatisation, as described by Lajmi et al. [35]. The amino acid content was expressed as the number of residues per 1000 residues.

### 3.9. Determination of GAGs Content

The absolute levels of GAGs were determined using a quantitative dye-binding (Alcian blue) method in the presence of chondroitin 4-sulfate as a standard [40]. The absorbance was measured at 600 nm by using a microplate reader, and the concentration of the GAGs was determined from a standard curve. The results were expressed as mg of chondroitin 4-sulfate per mg of dried hydrolysate.

### 3.10. Evaluation of the ACE-Inhibitory Activity

The ACE-inhibitory activity was measured according to Sentandreu and Toldrá [41], with some modifications. ACE was previously dissolved in 40 mL of 40% glycerol to reach a final concentration of 25 mU/mL. One unit of enzymatic activity was expressed as the amount of enzyme that produces 1 µmol of hippuric acid from HHL/min in 50 mM HEPES with 300 mM NaCl at pH 8.3 and at 37 °C. The assay was performed on a black 96-well plate. The initial determination of the ACE-inhibitory activity of the hydrolysates was performed using 40 μL (1 mU) of enzyme and 160 µL of the sample, previously diluted in the working buffer (150 mM Tris HCl buffer at pH 8.3 with 1.125 M NaCl) to reach a final concentration of 1 mg/mL in the system after addition of the substrate. The control samples contained 160 µL of the working buffer instead of the diluted sample. Blanks containing working buffer and ACE inactivated with 5 M HCl were also used. The microplate was incubated at 37 °C for 10 min. The reaction was initiated by adding 100 μL of the fluorogenic substrate, 0.45 mM Abz-Gly-Phe(NO_2_)-Pro, dissolved in the working buffer. The fluorescence generated was measured using the excitation and emission wavelengths of 360 nm and 400 nm, respectively, for 30 min at 1 min intervals, using a microplate reader. The inhibitory activity was calculated according to the following equation:

$$\mathrm{ACE}\;\mathrm{inhibitory}\;\mathrm{activity}\;\left(\%\right)=100-\left[\frac{S-B\times 100}{C-B}\right]$$

where C is the increment in fluorescence per minute from the control samples, S is the increment in fluorescence per minute from the samples, and B is the increment in fluorescence per minute from the blanks.

Different concentrations of the sample were used to calculate the IC_50_ values (sample concentration required to inhibit enzyme activity by 50%). The results were expressed as µg/mL.

### 3.11. Fractionation and Purification of Active Peptides

The freeze-dried hydrolysates (0.25 g) were fractionated using a preparative HPLC (Agilent LC PREP 1260 Infinity Series, Santa Clara, CA, USA). They were previously resuspended in 10 mL of Milli-Q water, filtered through 0.2 µm pore size filters, and then loaded onto a Europa protein C18 semi-preparative column (particle size 5 µm, 25 cm × 1 cm, Teknokroma, Barcelona, Spain). Fractions were collected at a flow rate of 2.5 mL/min. The solvents used were Milli-Q water (A) and acetonitrile (B), both with 0.1% trifluoroacetic acid (TFA). The gradients of B used were 0–5% (0–10 min), 5–58% (10–30 min), 58–58% (30–35 min) and 58–0% (35–45 min). The absorbance was measured at 215 nm. The fractions that showed the highest ACE-inhibiting activity were dried in a speed-vac, freeze-dried, resuspended in Milli-Q water. They were then separated by RP-HPLC (Shimadzu LC-10ADVP, Shimadzu, Kyoto, Japan) on a TRACER-Excel 120 ODS-A semi-preparative column (Teknokroma, Barcelona, Spain) with a particle size of 5 μm and dimensions of 25 cm × 0.78 cm. The solvents used were Milli-Q water (A) and acetonitrile (B), both with 0.1% TFA. Different gradients of acetonitrile-0.1% TFA in 30 min were used: A_IV_: 10–40%; A_V_: 10–45% and E_IV_: 15–45%.

The fractions showing the highest ACE inhibiting activity were selected, and their composition was obtained as follows.

### 3.12. Identification of the Peptide Composition of the ACE-Inhibiting Fraction

#### 3.12.1. Analysis by RP-LC-ESI-MS/MS

The peptides were analysed using an EASY-nLC 1000 liquid chromatograph (Thermo Scientific, Bremen, Germany) connected to a Q-Exactive HF hybrid quadrupole-Orbitrap mass spectrometer with a Nano-Easy spray source (ion transfer temperature of 250 °C and ion spray voltage of 1.8 Kv). The peptides were dried in a speed-vac and subsequently resuspended in 2% acetonitrile with 0.1% formic acid. They were then separated using a C18 Acclaim PepMap 100 Trapping precolumn (20 mm × 75 μm inner diameter, 3 μm, 100 Å pore size from Thermo Scientific) coupled with a C18 reverse phase analytical column Picofrit (500 mm × 75 μm inner diameter, 2 μm, 100 Å pore size from Thermo Scientific). The solvents used were ultrapure water with 0.1% formic acid (A) and 100% acetonitrile with 0.1% formic acid (B). The gradients of B used ranged from 2% to 40% for 30 min, at a constant flow rate of 250 nl/min. The Xcalibur 4.0 software was used for data processing. The data were recorded with the Full-MS data-dependent acquisition (DDA) method in positive mode. The MS1 scans were recorded in the 340–1600 Da m/z mass range and had a mass resolution of 60,000 Da and an automatic gain control (AGC) target of 3E6. The maximum ion time (ITmax) was 20 ms. The DDA method chose the top ten most prevalent precursors with charges from +2 to +6 in MS 1 scans for a higher-energy collisional dissociation (HCD) fragmentation. A dynamic exclusion of 15 s and an isolation mass window precursor of 2 m/z were used. The threshold for triggering the MS2 scans was 1.5 × 10^4^. The normalized collision energy (NCE) was 20%. The resolved fragments were scanned with a mass resolution of 30,000 Da, an AGC target value of 2 × 10^5^ and an ITmax of 200 ms.

#### 3.12.2. Analysis of MS/MS Spectra

The MS/MS data recorded on the Q-EXACTIVE HF were processed with Peaks Studio v 10.5 software (Bioinformatic solution Inc., Waterloo, ON, Canada) for peptide and protein identification, de novo peptide sequencing and analysis and characterization of post-translational modifications (PTM analysis). The search against the database was performed using Peaks and the following search parameters: parent mass error tolerance of 10 ppm, fragment mass error tolerance of 0.02 Da, precursor mass search type: monoisotopic, no enzyme, unspecific digest mode, max missed cleavages of 100, max variable PTM per peptide of 3 and enabled FDR estimation. The databases used were Uniprot (16,340 sequences from the collagen of actinopterygii and 436 sequences from proteins of the Triglidae family) and SwissProt (561,674 sequences without taxonomic restriction). The searches were carried out considering optional methionine oxidation (+15.99) and the hydroxylation of lysine or proline (+15.99). A contaminant database was also used. The maximum number of variable modifications per peptide was three. The decoy fusion approach, included in the software Peaks, was used to estimate the FDR (false discovery rate). An FDR ≤ 1% was selected to choose the peptides identified correctly. The acceptance criteria were: peptides with a −10 logP score ≥ 20 with at least one identified peptide.

The Peaks Studio 10.5 software was used to extract peptide sequences from the MS/MS Spectra without the need for a database. Peptides whose sequences did not exceed the −10lgP scores established to be confidently identified against the database were considered de novo sequences. They were screened by an average local confidence score (ALC) higher than or equal to 80. The ‘Peaks PTM’ module was also used to find possible unspecified modifications (up to 313) with a maximum of up to three per peptide, both in peptides identified against the database and in those obtained by de novo sequencing, provided that they exceeded a certain quality (ALCscore > 15). The total area of the precursor ions in the full MS scans was used to calculate the relative abundance.

### 3.13. In Silico Gastrointestinal Digestion of the ACE-Inhibiting Fractions

The stability of the peptides identified in the ACE-inhibitory fractions in the presence of gastrointestinal proteases was evaluated in silico, using the enzyme action tool in the BIOPEP-UWM database [42]. The enzymes used were Pepsin (pH 1.3), Trypsin and Chymotrypsin (A). The released peptides were analysed for the potential biological activity, also using BIOPEP-UWM.

### 3.14. Statistical Analysis

All trials were conducted in triplicate, and the mean was used to express the results. The data were subjected to analysis of variance (ANOVA), and Duncan’s multiple range test was used to check for differences between means. The Statistical Package for the Social Sciences (SPSS) version 10.0 (Chicago, IL, USA) was used to analyse the data. Differences were considered significant at *p* < 0.05.

## 4. Conclusions

The use of Alcalase or Esperase to hydrolyse tub gurnard skin protein without a previous protein extraction step releases very potent ACE-inhibitory peptides and GAGs that demonstrated antihypertensive potential. The ACE-inhibitory activity of the fractions appears to be due to the concomitant action of several peptides in a mechanism related to their hydrophobicity, as well as to the presence of specific residues in the last positions of the C-terminus. Although these peptides are likely to be degraded by gastrointestinal digestion, shorter ACE-inhibitory peptides with a higher potential for crossing the intestinal barrier would be released. The use of these fractions as functional ingredients would allow for the valorisation of the tub gurnard, considered a discard in Mediterranean countries. Further work should focus on studying the in vivo antihypertensive effect of the ACE-inhibitory fractions and GAGs, although the latter must be protected to prevent their gastrointestinal digestion. Likewise, the use of the protein muscle as raw material for the production of restructured products and the upgrading of other by-products such as heads, viscera and bones should be further explored as part of a comprehensive use of this abundant and underutilised fish species.

## Figures and Tables

**Table 1:** Chemical constituents and amino acid residue composition of the raw material (number of residues/1000 residues).

Amino Acids	‰
Asp + Asn (D + N)	67
Thr (T)	34
Ser (S)	56
Glu + Gln (E + Q)	85
Gly (G)	269
Ala (A)	111
Cys (C)	4
Val (V)	29
Met (M)	19
Ile (I)	17
Leu (L)	41
Tyr (Y)	10
Phe (F)	21
Hyl	7
His (H)	10
Lys (K)	38
Arg (R)	48
Pro (P)	80
Hyp	54
TEAA *	209
THAA *	587
Pro + Hyp	134

* TEAA = total essential amino acid residues: Σ Ile + Leu + Lys + Met + Phe + Thr + Val + His + Arg. * THAA = total hydrophobic amino acid residues: Σ Pro + Ala + Val + Met + Gly + Ile + Leu + Phe.

**Table 2:** Conditions summary of enzymatic hydrolysis, average molecular weight at 214 and 280 nm, and ACE-inhibitory activity of the different hydrolysates at a final concentration of 1 mg/mL (dried weight).

Enzyme	Enzymatic Conditions		MW at 214 nm (Da)	MW at 280 nm (Da)	ACE-Inhibition (%)
	pH	Temperature (°C)			
Esperase	8.5	55	729	358	93.92 ± 2.04 ^a^
Alcalase	8.5	55	927	545 (48%)	86.16 ± 2.29 ^b^
				375 (52%)	
Trypsin	8.5	40	2052	1646	90.92 ± 2.04 ^a^
Pronase E	8.5	55	3700	2165	83.34 ± 0.73 ^b^

Values for ACE inhibition are expressed as mean ± SD (n = 3). Values followed by different letters are significantly different (*p* < 0.05).

**Table 3:** ACE-inhibitory activity of the PRFs and GAGs at 1 mg/mL (dried weight).

Sample		ACE Inhibition	
		Inhibition (%)	IC_50_ Value (μg/mL)
PRFs	Esperase	91.44 ± 0.69 ^a^	47 ± 2
	Alcalase	91.12 ± 0.63 ^a^	68 ± 2
	Trypsin	89.37 ± 0.44 ^a^	89 ± 2
GAGs	Esperase	49.81 ± 2.32 ^d^	-
	Alcalase	52.34 ± 0.23 ^b^	-
	Trypsin	62.99 ± 2.09 ^c^	-

Values followed by different letters are significantly different (*p* < 0.05).

**Table 4:** Most abundant peptides identified in the most potent ACE-inhibiting fraction (A_IV-5_) derived from the Alcalase hydrolysate. P(+15.99) = Hydroxylation; M(+15.99) = Oxidation. Sub P= Mutation (S by P). Accession numbers, 1: A0A3Q4MFA6; 2: A0A291LSB7, G3NNJ9, G3NNI9, A0A3Q3WV51, A0A3Q3X9K2, A0A3Q3WV43, A0A3Q3X9H7, A0A2I4D9D9, A0A2I4D9D6, A0A4U5VUI1, A0A3S2Q3J5, A0A3Q2XU67, A0A3P9NHJ7, H3C3J9, H3C397; 3: A0A291LSB7, A0A3B4B451, A0A291LSN1; 4: A0A3B4B451, A0A3Q2FX51.

Peptide	−10lgP	Mass (Da)	ppm	ALC (%)	Relative Abundance (%)	Parental Protein
(1) GPPGS(sub P)PGPAGPP(+15.99)GPPGSGM	23.48	1586.7	−0.2		37.5	Collagen (1)
(2) GM(+15.99)P(+15.99)GERGAAGLP(+15.99)GLR	23.16	1485.7	1.1		24.6	Collagen (2)
(3) GPAGIVGPP(+15.99)GPAGPA	28.06	1229.6	1.6		9.9	Collagen (3)
(4) GVEDELDKY		1066.5	−0.6	97	4.5	
(5) VNPVYEGY		939.4	0.6	86	4.0	
(6) GPP(+15.99)GSP(+15.99)GLPGPPGPA	23.71	1285.6	1.8		1.7	Collagen (4)
(7) TDGLDGPYDELK		1321.6	−0.5	90	1.0	
(8) TGFPDPVAGN		973.4	0.2	85	1.0	
(9) TGFPPDVAGN		973.4	0.2	86	1.0	
(10) TGFPPVDAGN		973.4	0.2	89	1.0	
(11) TGFPPDVAGN		973.4	0.2	94	1.0	
(12) SADAPM(+15.99)FVM(+15.99)		999.4	−1.2	94	0.9	
(13) QSLFPLQ		831.4	0.1	82	0.9	

**Table 5:** Most abundant peptides identified in the most potent ACE-inhibiting fraction (E_IV-1_) derived from the Esperase hydrolysate. P(+15.99) = Hydroxylation; K(+14.02) = Methylation. Accession numbers: 1: A0A3Q3NAA8, A0A484CKF1, A0A484CIB2, A0A3Q2Z8D3, A0A3Q1J299, Q5NT95, G3NNI9, E6ZHW3, A0A3Q3GSY4, A0A3P8VXI7, A0A3B3YTU0|, 0A3B4B451, A0A3Q4MFA6, A0A3Q4GXS1, A0A3Q3KIB6, A0A2I4D9D9, A0A2I4D9D6, A0A2I4D9D4, A0A3Q1D832, A0A3P8TJL5, A0A3Q2QLQ8, G3ND57, G3ND40, G3ND52, A0A3Q2PWY4, H3DDS5, H3C3J9, H3C397, A0A3Q4BWQ8, A0A484CW52, A0A3Q0QS39, Q5NT96, A0A3Q1ERK6, H2LEV0, A0A3P9M3E0, A0A3S2M1M2, A0A0S7F2G1, A0A0S7F6B4, A0A096M208, A0A087YA18, A0A3P9QC20, A0A3P9QBP9, A0A0S7F0V8, A0A0S7FB16, A0A0S7FAY2, A0A0S7F0W1, H3D7E7, H3CKB6, H3C6Q3, G9M6I6, I3JXH5, A0A3P8R1C1, A0A3P9BM02, A0A3P8R2U1, I3JXH4, A0A3P8W379, A0A087Y5A9, A0A3B4ZMN5, A0A3B4TSF4, A0A3B4XD70, A0A3P8UX40, A0A4U5VEZ8, A0A0S7GW03, A0A087YCK5, A0A3B3Y6G8, A0A146V1L1, A0A3B3U6U6, A0A3Q3NRA6, A0A0F8C484, A0A3Q1FVW0, A0A3B5B9F6, A0A437CWD9; 2: H3DDS5, H3C3J9, H3C397; 3: A0A484CKF1, A0A3Q1J299, Q5NT95, A0A3P8VXI7, A0A3Q3KIB6, A0A3Q1D832, A0A3P8TJL5; 4 and 6: A0A3Q3NAA8; 5: A0A484CKF1, A0A3Q1J299|Q5NT95, A0A3P8VXI7, A0A3Q3KIB6, A0A3Q1D832, A0A3P8TJL5; 7: A0A3Q3NAA8, A0A484CKF1, Q5NT95, A0A3P8VXI7, A0A3B4B451, A0A3Q3KIB6, A0A3Q4BWQ8, A0A3Q0QS39, G9M6I6, I3JXH5, A0A3P8R1C1, A0A3P9BM02, A0A3P8R2U1|I3JXH4; 8: A0A3Q1D832, A0A3P8TJL5.

Peptide	−10lgP	Mass (Da)	ppm	ALC (%)	Relative Abundance (%)	Parental Protein
(1) LLAPPERKY		1085.6	−0.7	86	30.84	
(2) ELEEELEAE		1089.5	−1.6	94	15.44	
(3) GPRGPAGPL		820.4	1.2	92	11.89	Collagen (1)
(4) SLDDKVELE		1046.5	0.8	96	8.49	
(5) KYPLEHALLTK		1311.8	−1.8	84	5.80	
(6) AGPP(+15.99)GP(+15.99)AGPP(+15.99)APGAPGGGFD	23.2	1690.8	1.6		2.17	Collagen (2)
(7) AGPP(+15.99)GFP(+15.99)GGPGP(+15.99)KGEIGPA	27.77	1706.8	1.5		2.00	Collagen (3)
(8) AGPP(+15.99)GFP(+15.99)GGPGP(+15.99)KGELGPA	27.77	1706.8	1.5		2.00	Collagen (4)
(9) AGP(+15.99)PGFP(+15.99)GGP(+15.99)GPKGEIGPA	27.46	1706.8	1.5		2.00	Collagen (5)
(10) AGP(+15.99)PGFP(+15.99)GGP(+15.99)GPKGELGPA	27.46	1706.8	1.5		2.00	Collagen (6)
(11) LEHEEGKLL		1066.6	−1.9	98	1.77	
(12) KTDDGKLFT		1023.5	−1.5	90	1.00	
(13) QP(+15.99)GNTGLPGMT		1087.5	2	91	0.99	Collagen (7)
(14) NIGFP(+15.99)GPK(+14.02)GASGDPGKP(+15.99)GDKGATGPS	24.18	2411.1	−0.6		0.98	Collagen (8)

**Table 6:** ACE-inhibitory peptides released by in silico gastrointestinal digestion of the peptides in fraction A_IV-5_. The results were obtained using the enzyme action tool of the BIOPEP-UWM database.

Sequence	Mass (Da)	Times Released
GY	238	1
PGL	285	2
PL	228	1
AF	236	1
GL	188	1
DF	280	3
VM	248	2

**Table 7:** Peptides released by in silico gastrointestinal digestion of the peptides in fraction E_IV-1_. The results were obtained using the enzyme action tool of the BIOPEP-UWM database.

Sequence	Mass (Da)	Times Released
GY	238	3
PL	228	5
IW	317	1
VK	245	1
GM	206	2
GL	188	1
GK	203	2
VR	273	1
EK	275	1
TF	266	1
AGDDAPR	700	1
VM	248	1
ER	303	1

## Data Availability

Data are available upon request.

## Supplementary Materials

The following supporting information can be downloaded at: https://www.mdpi.com/article/10.3390/md21020131/s1, Figure S1: Elution profiles of PRFs derived from Alcalase and Esperase hydrolysates. Figure S2: Elution profiles of PRFs that showed the most potent ACE-inhibiting activity, at 214 nm and 280 nm. Table S1: ACE-inhibitory activity of fractions obtained by chromatography at a final concentration of 0.12 mg/ml (dried weight). Table S2: ACE-inhibitory activity of sub-fractions obtained by chromatography at a final concentration of 13 µg/ml (dried weight) and IC_50_ values of those showing the most potent activity.
