# Supplementary material for: Identification of ACE I-Inhibitory Peptides Released by the Hydrolysis of Tub Gurnard (Chelidonichthys lucerna) Skin Proteins and the Impact of Their In Silico Gastrointestinal Digestion
Source: Mar Drugs. 2023 Feb 17;21(2):131. doi: 10.3390/md21020131 (PMC9967738; doi:10.3390/md21020131)
Supplement: Supplementary file 1 [file marinedrugs-21-00131-s001.zip › marinedrugs-2212979-supplementary.pdf]

Figure S1. Elution profiles of PRFs derived from Alcalase (a) and Esperase (b) hydrolysates. The fractions were collected at different elution times (in minutes): a) 6 (A_{I}), 7 (A_{II}), 8-12 (A_{III}), 19-22 (A_{IV}), 23-30 (A_{V}); b) 6-8 (E_{I}), 18-19 (E_{II}), 20-22 (E_{III}), 23-25 (E_{IV}).

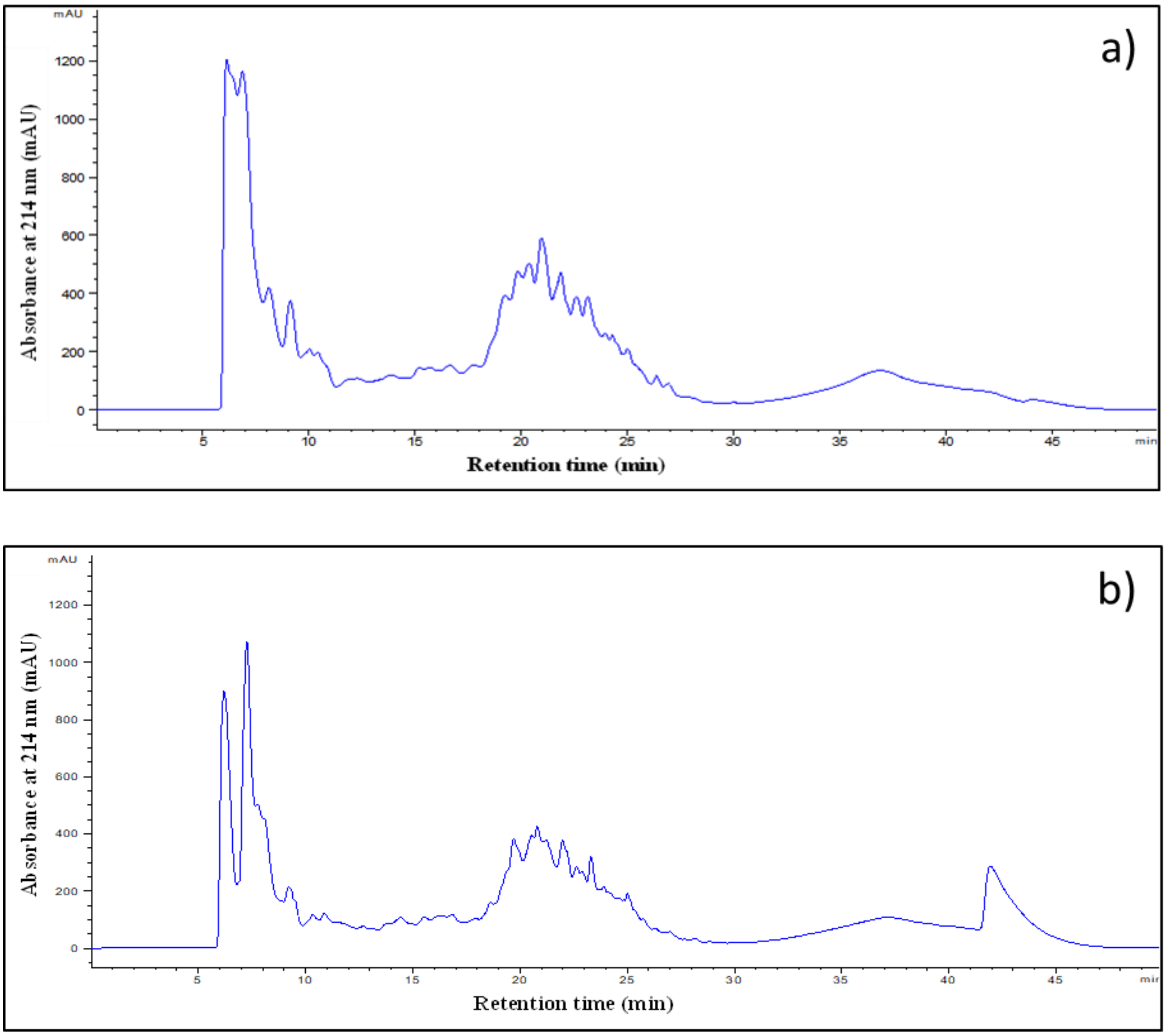

Figure S2. Elution profiles of PRFs that showed the most potent ACE-inhibiting activity, at 214 nm (in red) and 280 nm (in green). The fractions were collected at different elution times (in minutes): A_{IV}: 9.5-11.5 (A_{IV-1}), 11.5-12 (A_{IV-2}), 12-14,5 (A_{IV-3}); 14.5-15.5 (A_{IV-4}), 15.5-17 (A_{IV-5}), 17-19,5 (A_{IV-6}); A_{V}: 15.5-17.5 (A_{V-1}), 17.5-19.5 (A_{V-2}), 19.5-21.5 (A_{V-3}), 21.5-27 (A_{V-4}); E_{IV}: 12-13 (E_{IV-1}), 13-15 (E_{IV-2}), 15-16 (E_{IV-3}), 16-19 (E_{IV-4}), 19-20 (E_{IV-5}).

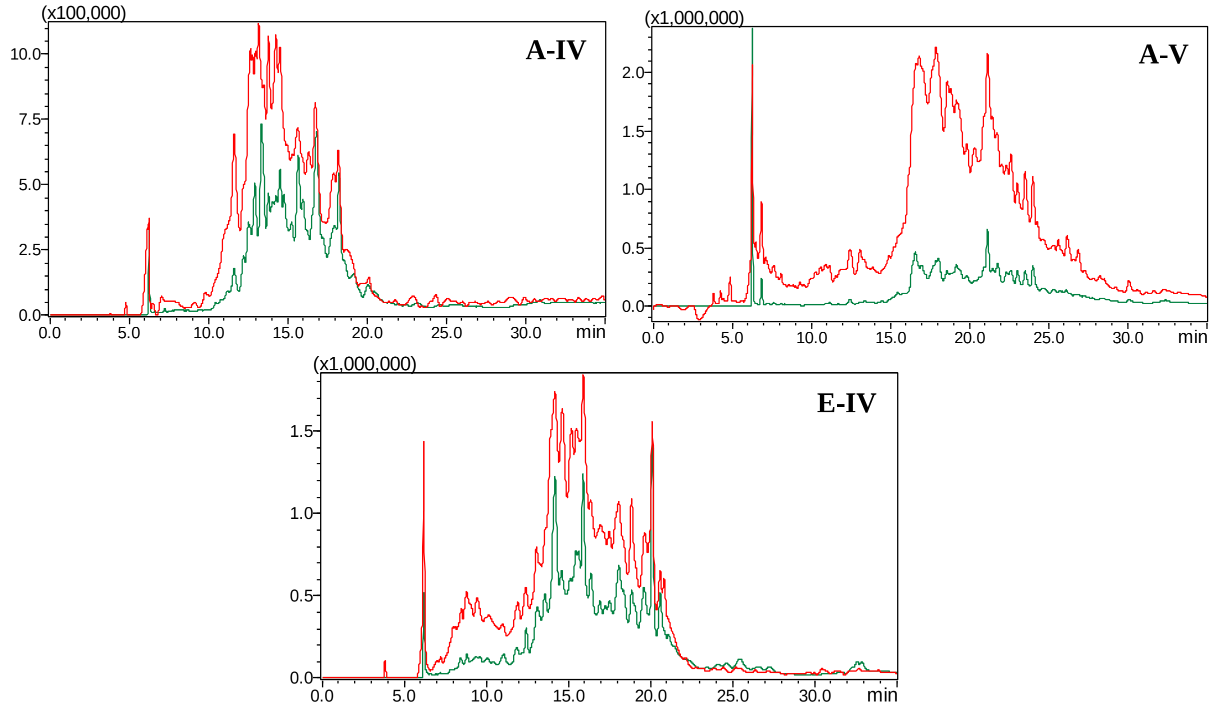

Table S1: ACE-inhibitory activity of fractions obtained by chromatography at a final concentration of 0.12 mg/ml (dried weight).

HPLC Sub-fractions		ACE inhibition (%)
**Esperase-PRF**	E_I	86.59±0.48
	E_{II}	85.33±2.01
	E_{III}	84.32±0.43
	E_{IV}	93.25±3.06
**Alcalase-PRF**	A_I	35.93±1.99
	A_{II}	52.69±0.80
	A_{III}	70.37±0.78
	A_{IV}	84.25±0.65
	A_V	86.06±0.30

Table S2: ACE-inhibitory activity of sub-fractions obtained by chromatography at a final concentration of 13 µg/ml (dried weight) and IC_{50} values of those showing the most potent activity.

HPLC Sub-fractions		ACE inhibition (%)	IC_{50} value (µg/ml)
E_{IV}	E_{IV-1}	23.17±2.37	33±3
	E_{IV-2}	No activity	
	E_{IV-3}	16.86±0.75	
	E_{IV-4}	10.56±3.77	
	E_{IV-5}	No activity	
A_{IV}	A_{IV-1}	7.90±3.40	
	A_{IV-2}	38.46±0.23	
	A_{IV-3}	6.01±0.85	
	A_{IV-4}	14.47±2.56	
	A_{IV-5}	44.95±2.35	29±6
	A_{IV-6}	No activity	
A_{V}	A_{V-1}	16.30±0.15	
	A_{V-2}	2.86±0.67	
	A_{V-3}	11.06±2.34	
	A_{V-4}	2.86±0.53	
